# Minimally displaced acetabulum fractures in geriatric patients: a comparison of open, percutaneous and non-operative treatment from the German Pelvic Injury Register data

**DOI:** 10.1007/s00068-020-01346-9

**Published:** 2020-04-08

**Authors:** Helene Ernstberger, Philipp Pieroh, Andreas Höch, Christoph Josten, Steven C. Herath, Georg Osterhoff, Working Group on Pelvic Fractures of the German Trauma Society

**Affiliations:** 1 Department of Orthopaedics, Trauma and Plastic Surgery, University Hospital Leipzig, 04103 Leipzig, Germany; 2 Department of Trauma, Hand and Reconstructive Surgery, Saarland University Hospital, Building 57, Kirrbergerstr. 1, 66421 Homburg, Germany

**Keywords:** Acetabulum fracture, Elderly, Percutaneous fixation, ORIF, Conservative treatment, Reduction

## Abstract

**Purpose:**

In elderly patients with minimally displaced acetabulum fractures, the patients’ inability to partially weight-bear and the need for early mobilisation may trigger the decision towards a treatment with higher primary stability. The purpose of this study was to compare open reduction and internal fixation (ORIF), closed reduction and percutaneous fixation (CRPIF) and non-operative treatment in geriatric minimally displaced acetabulum fractures with regard to complications and quality of reduction.

**Methods:**

Data from the prospective German Pelvic Injury Register collected between 2008 and 2018 were used to evaluate 608 geriatric patients with isolated minimally displaced (≤ 5 mm) acetabulum fractures. In total, 429 received non-operative treatment, 117 ORIF and 62 CRPIF. Demographics, injury severity, fracture pattern, complications and fracture displacement before and after treatment were analysed.

**Results:**

Both operative methods reduced fracture gap displacement. CRPIF was associated with lower blood loss and shorter operative time compared to ORIF (*p* < 0.001). Hospital stay was 12.9 days in the non-operative group, 16.8 with CRPIF and 23.6 with ORIF (*p* < 0.001). Non-surgical general complications were more likely to occur following ORIF (22.2%) compared to CRPIF (8.1%) and non-operative treatment (8.4%, *p* < 0.001). The rate of surgical complications was not different for ORIF and CRPIF (*p* = 0.122)

**Conclusion:**

Both operative treatments improve fracture displacement and joint congruency in elderly patients with minimally displaced acetabulum fractures. Compared to ORIF, CRPIF achieves similar quality of reduction but is associated with fewer complications, smaller intraoperative blood loss, shorter operative time and shorter length of hospital stay.

## Introduction

The incidence of acetabular fractures in the elderly has increased by 2.4-fold since 1980 [1] and represents the most rapidly growing group within acetabular injuries [2]. Many studies have outlined the differences of acetabulum fractures between young and old ages [1, 3]. The mechanism of injury in young patients is mainly high-energy trauma, more frequently affecting the posterior parts of the acetabulum. In the geriatric group, the trauma is most commonly a simple fall and more likely to be associated with anterior column fractures [1, 3]. Consequently, different standards of treatment should apply for the elderly than those used in young ages.

Treatment options include conservative treatment, open reduction and internal fixation (ORIF) and closed reduction and percutaneous fixation (CRPIF). Minimally displaced acetabular fractures usually can be considered stable and thus be treated non-operatively [3, 4]. A recent study even showed good functional outcome for acetabulum fractures with greater displacement and non-operative treatment in the elderly [5]. However, as prolonged bed rest is associated with increased morbidity and mortality [6], early mobilisation is essential [7]. ORIF allows for direct visualization of the fragments and has the potential to result in a good quality of reduction and early mobilisation [3]. In young patients, better reduction is associated with a better clinical outcome and a lower rate of secondary osteoarthritis [3, 8–12]. Many studies identified higher age as a negative predictor regarding reduction, mortality and complications, though [1, 13–15]. Percutaneous screw fixation of acetabulum fractures has the advantage of less invasive approaches and lower blood loss and, hence, seems to be a potential alternative for geriatric patients [15–17]. However, percutaneous techniques only allow for closed reduction and may result in inferior fracture reduction when compared to ORIF. Which treatment is best for elderly patients is still under debate.

The aim of this study was to compare open operative, percutaneous operative and non-operative treatment of acetabulum fractures in geriatric patients with regard to non-surgical general and surgical complications, intraoperative blood loss, and quality of reduction achieved.

## Materials and methods

A retrospective multicentre registry study was conducted analysing the register of the German pelvic work group (Pelvic Injury Register of the German Trauma Society), as part of the German Society of Traumatology (DGU) and German section of the AO Trauma.

This study was approved by the local institutional ethics committee (Ethik-Kommission Leipzig, 119/19-ek).

### Patients

In total, 3432 patients with acetabulum fractures were documented in the German Pelvic Trauma Registry from 07/2008 until 03/2018. Patients aged 60 years and older with a preoperative gap/step of ≤ 5 mm were included. Within the inclusion criteria, 829 patients were eligible.

Patients with combined acetabulum and pelvic ring fractures of type B/C (tile classification [18, 19]) or sacral fractures (*n* = 156), with bilateral acetabular fractures (*n* = 16), periprosthetic fractures (*n* = 33) fractures due to metastatic disease (*n* = 4) and those treated with primary total hip arthroplasty (*n* = 10) were excluded. In addition, we had to exclude two patients receiving THA half a year after the injury, as information about initial treatment for the fracture was missing. Finally, 608 patients were analysed for this study. Patients were assigned to three groups: open reduction and internal fixation (ORIF, *n* = 117), closed reduction and percutaneous internal fixation (CRPIF, *n* = 62), and non-operative treatment (*n* = 429). For further analysation, patients were separated in two groups: 60–79-year-old (*n* = 349) and 80–100-year-old (*n* = 259) patients.

### Data acquisition

All data were extracted from the central database of the Pelvic Injury Register of the German Trauma Society. For statistical analyses, data were selected and transferred into a SPSS table. This information included age and gender, fracture pattern according to Judet and Letournel [3], hospital duration and the treatment method. Based on plain radiography (anteroposterior pelvic view), the fracture gap and step had been evaluated before and after surgery. Complications that could be directly linked to the surgical procedure were documented as “surgical complications”. Complications that occurred during the hospitalisation but without direct reference to the surgical procedure were documented as “non-surgical general complications”. For the operative treatment groups, operation duration and intraoperative blood loss were documented. Patients were followed up until their day of discharge.

### Statistical analysis

Statistics were performed using SPSS 24.0 (SPSS Inc., Chicago, IL, USA). Continuous data are presented as mean with standard deviation (SD), categorical data as absolute number of cases (*n*) and percentage (%).

Primary outcome was the occurrence of general complications during hospital stay and surgical complications. A Pearson Chi-square test was used to assess differences regarding complication rates between the three groups and between 60–79 and 80–100-year-old patients. Likewise, a Chi-square test was performed to compare the two surgical methods concerning surgical complications. As secondary outcome, we analysed the hospital duration, quality of reduction, intraoperative blood loss and operative time. Metric, non-Gaussian variables, in our case age, ISS score, hospital duration and fracture gap/step were evaluated performing a Kruskal–Wallis *H* test. Concerning paired comparison, post hoc tests with Dunn–Bonferroni correction were executed. A Mann–Whitney *U* test was chosen to detect differences regarding intraoperative blood loss, since the variable exhibited non-normal distribution, and fracture gap/step regarding age. As for 12 patients treated openly, a blood loss of 0 ml was documented, these patients had to be excluded analysing this variable. The operative time was evaluated using a Student’s *T* test. A *p* value of < 0.05 was considered as statistically significant. Boxplots were prepared to illustrate changes of fracture gap/step using operative treatment and in comparison to non-operative treatment.

## Results

### Patients’ baseline characteristics

The mean age at time of the trauma was 77.3 (SD 9.7) years (range 60–100), 227 (37.3%) were of female gender (Table 1). The mean injury severity score (ISS) was 10.9 (SD 5.79), showing similar scores in all three groups (*p* = 0.546).

**Table 1:** Patients’ baseline characteristics

	Treatment				
	Non-operative	Percutaneous	Open	*p*	Total
*N*	429	62	117		608
Age (years)	78.7 (9.71)	74.3 (10.02)	74.1 (8.08)	0.000^˩^	77.3 (9.66)
Gender (f:m)	185:244	12:50	30:87		227:381
Fracture gap after trauma (mm)	1.59 (1.26)	2.47 (1.46)	3.11 (1.57)	0.000^˩^	1.97 (1.48)
Fracture step after trauma (mm)	0.97 (1.20)	1.32 (1.38)	2.37 (1.54)	0.000^˩^	1.28 (1.40)

^˩^Kruskal–Wallis *H* test, SD in brackets

Patients receiving non-operative treatment were significantly older than patients treated by CRPIF (*p* = 0.004) or ORIF (*p* < 0.001), whereas patients who were treated by CRPIF or ORIF were not different in age (*p* = 1.00). The most common fracture pattern according to Judet and Letournel [3] was an isolated anterior column fracture (*n* = 191; 31.4%), followed by combined anterior column posterior hemitransverse (*n* = 123; 20.2%) and anterior wall fractures (*n* = 94; 15.5%, Table 2).

**Table 2:** Fracture patterns

	Treatment			
	Non-operative	Percutaneous	Open	Total
N	429	62	117	608
Fracture pattern				
PW	32 (7.5%)	0	8 (6.8%)	40 (6.6%)
PC	20 (4.7%)	2 (3.2%)	4 (3.4%)	26 (4.3%)
AW	88 (20.5%)	3 (4.8%)	3 (2.6%)	94 (15.5%)
AC	141 (32.9%)	30 (48.4%)	20 (17.1%)	191 (31.4%)
Transverse	23 (5.4%)	4 (6.5%)	2 (1.7%)	29 (4.9%)
PW/PC	1 (0.2%)	0	3 (2.6%)	4 (0.7%)
Transverse/PW	4 (0.9%)	1 (1.6%)	7 (6.0%)	12 (2.0%)
T shaped	27 (6.3%)	2 (3.2%)	4 (3.4%)	33 (5.4%)
ACPHT	71 (16.6%)	15 (24.2%)	37 (31.6%)	123 (20.2%)
Both columns	15 (3.5%)	4 (6.5%)	28 (23.9%)	47 (7.7%)
Unknown	2 (0.5%)	0	0	2 (0.3%)
No classification	5 (1.2%)	1 (1.6%)	1 (0.8%)	7 (1.2%)

*PW* posterior wall, *PC* posterior column, *AW* anterior wall, *AC* anterior column, *ACPHT* anterior column posterior hemitransverse

### Complications

Non-surgical complications were documented for 67 (11.0%, Table 3) patients. There was no significant difference between patients treated by CRPIF (*n* = 5; 8.1%) and non-operatively treated patients (*n* = 36; 8.4%, *p* = 0.931). With ORIF, however, 22.2% (*n* = 26) showed complications, which was a significantly higher rate compared to the other groups (CRPIF: *p* = 0.017; non-op.: *p* < 0.001). Concerning all patients, the most common complication documented was haematoma (*n* = 10). There were two patients with thrombosis, five with pulmonary embolism, three with acute respiratory distress syndrome (ARDS), two with multi-organ failure, seven neurologic complications, four superficial and seven deep wound infections, four bleedings, one secondary displacement and one case with delayed wound healing. For 36 patients with different complications from those mentioned above, “other non-surgical complications” were documented, summarised in Table 4. As three patients had more than one “other non-surgical complication” there are 36 patients, but 42 complications listed in Table 4. From the registry data available, it was not obvious what caused the infections, bleedings, drop of haemoglobin and anaemia and whether they occurred due to surgery or other injuries. Therefore, they were assigned to “non-surgical general complications”. Infections and bleedings with known relation to the surgical procedure were documented as “surgical complications”. Surgical complications included deep infection (*n* = 4), intraoperative bleeding (*n* = 2), implantat malpositioning or loosening with (*n* = 3) and without (*n* = 2) re-osteosynthesis and six “other complications” (Table 4). Surgical complications occurred in 3/62 cases with CRPIF (4.8%) compared to ORIF (14/117, 12.0%) but the difference was not significant (*p* = 0.122). In total, 17 patients (9.5%) had surgical complications (Table 3).

**Table 3:** Outcome

	Treatment				
	Non-operative	Percutaneous	Open	*p*	Total
*N*	429	62	117		608
OR duration (min)	–	91 (52)	169 (67)	0.000^†^	142 (73)
Blood loss (ml)^×^	–	73 (184)	639 (451)	0.000^‡^	429 (464)
Non-surgical complications	36 (8.4%)	5 (8.1%)	26 (22.2%)	0.000*	67 (11.0%)
Surgical complications	–	3 (4.8%)	14 (12.0%)	0.122*	17 (9.5%)
Hospital duration (days)	12.9 (12.8)	16.8 (12.0)	23.6 (15.2)	0.000^˩^	15.3 (13.8)
Fracture gap after treatment (mm)	–	1.2 (1.3)	1.3 (1.0)	1.00^˩^	1.2 (1.1)
Fracture step after treatment (mm)	–	0.5 (1.0)	0.8 (1.0)	0.844^˩^	0.7 (1.0)

× 12 patients in the ORIF group had to be excluded due to blood loss of 0 ml

*OR* operation room

*Pearson Chi-square test

^†^Student’s *T* test

^‡^Mann–Whitney *U* test

^˩^Kruskal–Wallis H test, SD and percentage in brackets

**Table 4:** Other complications

	Non-operative	Percutaneous	Open
Other non-surgical complications			
Pulmonary oedema		1	
Atelectasis			1
Pneumonia	3	1	1
Pulmonary aggravation	1		
Haemothorax	1		
Cardiac decompensation			1
Pacemaker due to cardiac arrythmia			1
NSTEMI	1		
Subacute myocardial ischemia	1		
Postoperative anaemia			1
Drop of haemoglobin			1
Urinary tract infection	3	1	1
Renal failure	1		
Renal failure of the transplanted renal	1		
Secondary necrosis of the femoral head			1
Acute hepatitis	1		
Sub ileus	1		
Caecum perforation			1
Pneumoperitoneum with diagnostic laparotomy			1
Perianal bleeding	1		
MRSA skin			1
Fall with laceration of the finger and face	1		
Decubitus	3		
Delir	1		
TIA	1		
Seizure	1		
Loss of consciousness	1		
TEP loosening of the other hip	1		
Free joint body			1
Pain	1		
TUR prostate	1		
Carbon dioxide narcosis	1		
Other surgical complications			
No reduction		1	
Dorsal extra osseous screw			1
Broken intra-articular K-wire			1
Postoperative bleeding (2 times)			1
Postoperative haematoma			1
Caecum perforation			1

*MRSA* multi-resistant *Staphylococcus aureus*, *TIA* transient ischemic attack, *TEP* total endoprosthesis, *TUR* transurethral resection

Comparing 60–79-year-old patients with 80–100-year-old patients, there was no significant difference regarding non-surgical complications (60–79: 38 (10.9%); 80–100: 29 (11.2%); *p* = 0.904) and surgical complications (60–79: 11 (8.3%); 80–100: 6 (8.7%); *p* = 0.384). However, after receiving CRIPF, no patient aged 60–79 had a surgical complication (0/43 cases; 0%) but 3 out of 19 patients aged 80–100 (15.8%) (*p* = 0.026).

### Length of operation and hospitalisation, and intraoperative blood loss

The mean hospital stay length was 15.3 days (SD 13.8, Table 3). Non-operative treatment was associated with the shortest mean hospital stay, followed by CRPIF (*p* < 0.001) and ORIF (*p* < 0.001). To measure the influence of the different follow-up times, a partial correlation was being performed, showing that the complication rate after ORIF was no longer higher than after CRPIF and non-operative treatment (*p* = 0.758).

Both, the mean operation time (*p* < 0.001) and intraoperative blood loss (*p* < 0.001) were significantly lower when the surgery was performed percutaneously (Table 3).

### Quality of reduction

**Fig. 1:**
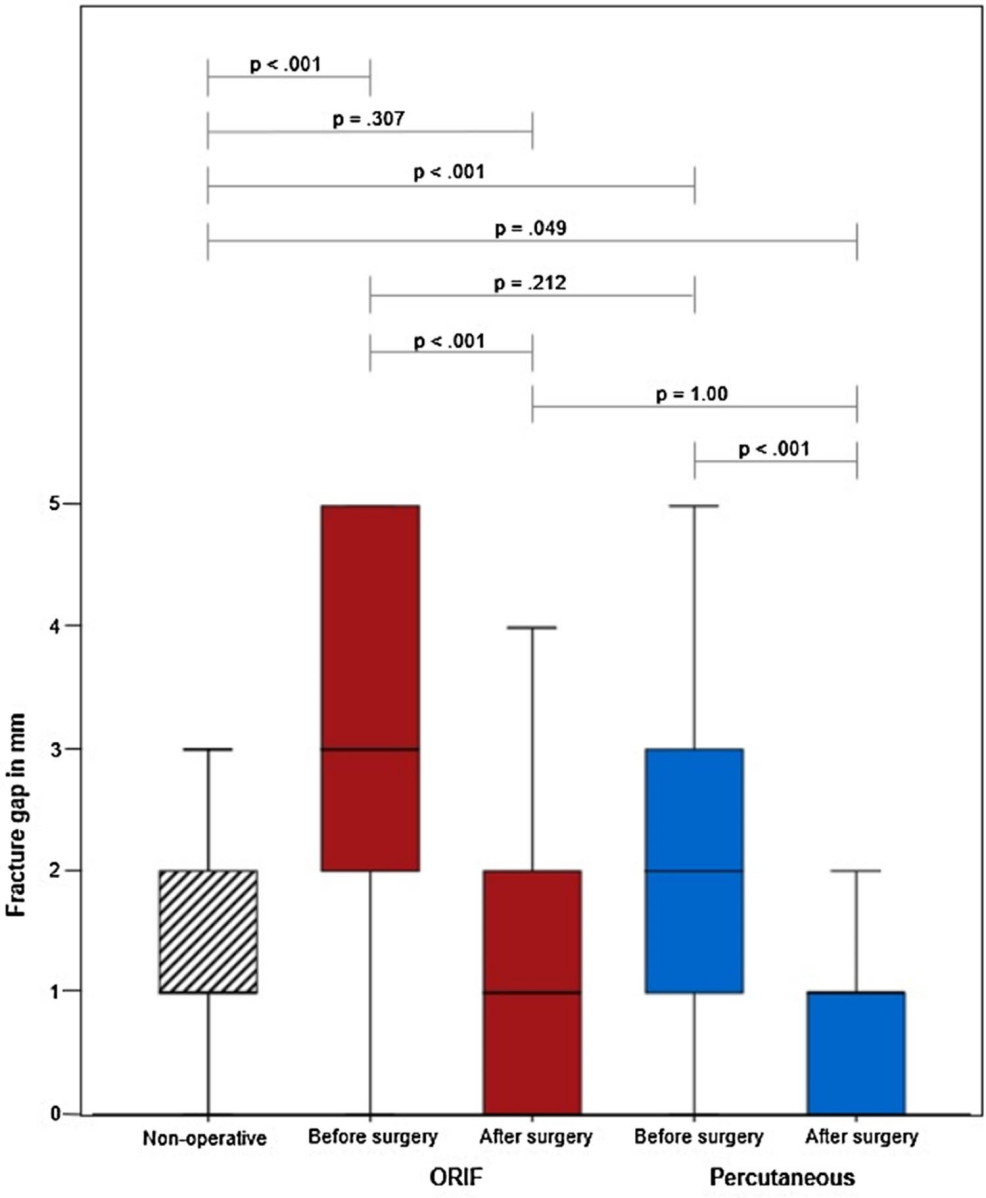
Fracture gap of minimal displaced acetabulum fractures. Comparison of fracture gap in mm of open reduction and internal fixation (ORIF) before and after surgery (red), percutaneous treatment before and after surgery (blue) and non-operative treatment (black stripes). The boxes show the percentiles 25, 50 (Median) and 75. The end of the whiskers shows 1.5 × interquartile range. Outliers and extremes are not shown. A Kruskal–Wallis *H* test with Dunn–Bonferroni correction was performed

**Fig. 2:**
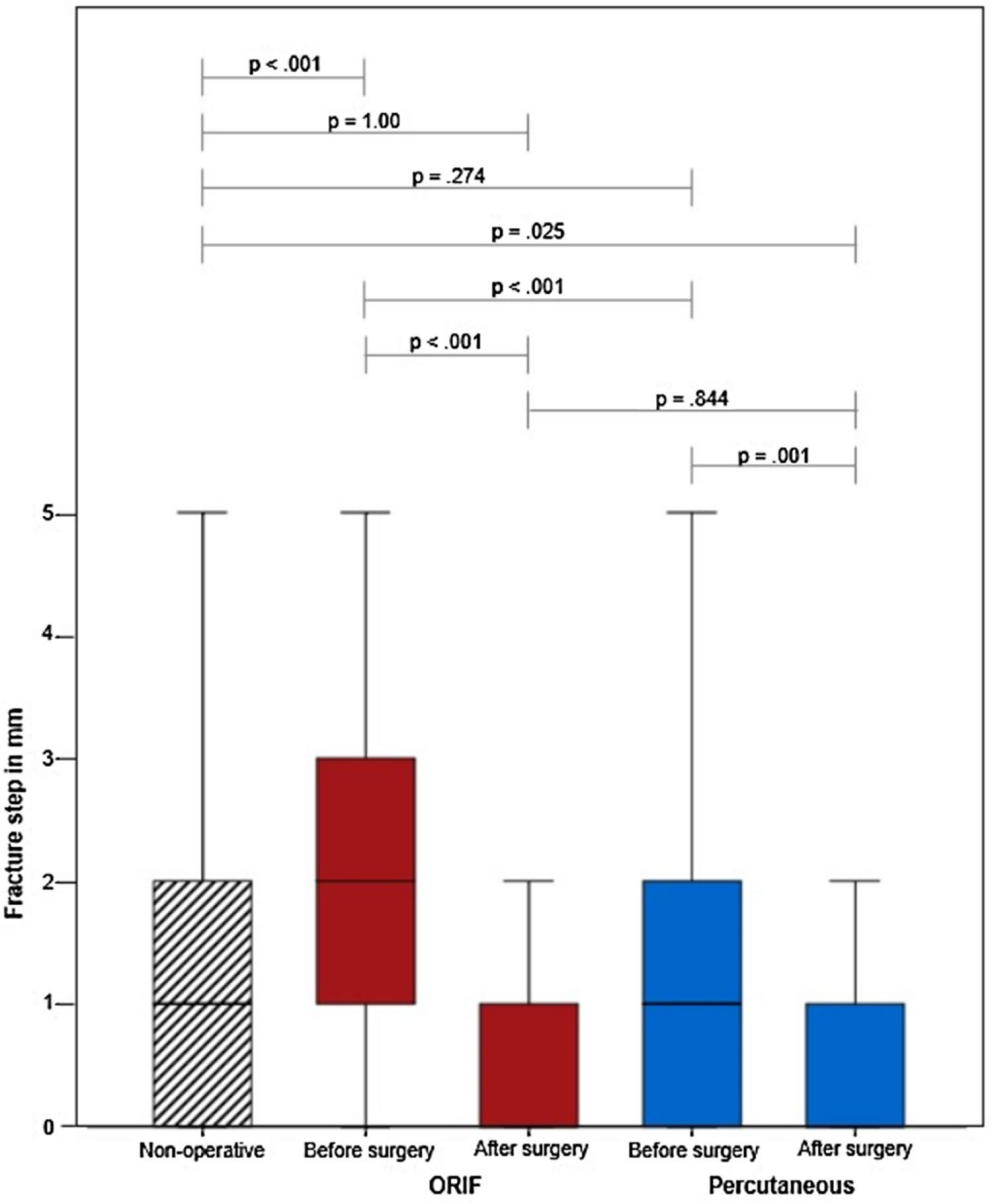
Fracture step of minimal displaced acetabulum fractures. Comparison of fracture step in mm of open reduction and internal fixation (ORIF) before and after surgery (red), percutaneous treatment before and after surgery (blue) and non-operative treatment (black stripes). The boxes show the percentiles 25, 50 (Median) and 75. The end of the whiskers shows 1.5 × interquartile range. Outliers and extremes are not shown. A Kruskal–Wallis *H* test with Dunn–Bonferroni correction was performed

Mean fracture gap after trauma was 2.0 mm (1.5 SD), mean fracture step 1.3 mm (1.4 SD). Patients treated non-operatively had a significant lower mean posttraumatic articular gap compared to patients where the decision for open (*p* < 0.001) or percutaneous (*p* < 0.001) surgery had been made (Table 1, Figs. 1 , 2). The mean posttraumatic, pre-treatment articular step in the non-operative group was similar to that seen in the CRPIF group (*p* = 0.274) but smaller compared to the ORIF group (*p* < 0.001).

Both operative methods achieved significant articular fracture gap and step reduction (*p* < 0.05, Figs. 1, 2) when comparing postoperative to posttraumatic displacement. Concerning quality of reduction, no difference could be found between percutaneous and open treatment (gap: *p* = 1.00; step: *p* = 0.844). Percutaneous fixation reduced fracture step/gap to smaller dimensions than those seen in non-operatively treated patients (gap: *p* = 0.049; step: *p* = 0.025).

Comparing 60–79-year-old patients with 80–100-year-old patients, no significant difference can be seen regarding articular displacement in the non-operative group (gap: *p* = 0.068; step: *p* = 0.153) after ORIF (gap: *p* = 0.725; step: *p* = 0.361) and after CRPIF (gap: *p* = 0.083; step: *p* = 0.419). However, in patients aged 80–100, fracture gap and step could not be reduced significantly using CRPIF (gap: *p* = 0.134; step: *p* = 1.00), but using ORIF (gap: *p* < 0.001; step: *p* < 0.001).

## Discussion

The optimal treatment of minimally displaced acetabulum fractures in the elderly remains controversial. The advantage of better primary stability with operative fixation has to be weighed against the potential complications of a surgical intervention. Surgery, however, can be performed in different degrees of invasiveness. This study compared ORIF, CRPIF and non-operative treatment regarding non-surgical and surgical complications, intraoperative blood loss, and quality of reduction.

The fracture patterns seen in this study were mainly those affecting the anterior column of the acetabulum. These are the common patterns described for geriatric patients and one markedly difference between old and young individuals [1, 3, 20, 21]. It underlines even more the need for research aimed specifically for geriatric acetabulum fractures.

In this study, using any of the two operative methods achieved significant reduction of articular fracture displacement. Many studies have underlined optimal reduction as an important factor for good clinical outcome and prevention of late complications [4, 9–13, 22, 23]. Regardless of age, Matta et al. highlighted the importance of fracture reduction to at least 3-mm gap and step [24]. Verbeek et al. described a cut-off at 5-mm gap and 1-mm step, others mentioned a cut-off at 2-mm gap and step as a measure for a good outcome [4, 25]. The mean values that could be achieved by operative treatment in this study remain below these thresholds. This might be somewhat unexpected, since several authors seemed to expect worse outcome for percutaneous treatment [17, 26, 27]. Most of these studies are old and, as minimal invasive treatment is developing, better results can be expected now [28–30]. However, studies comparing quality of reduction are rare. To the authors’ knowledge, only Jeffcoat et al. compared an open and limited ilioinguinal approach with the result of equivalent reduction for both methods, similar to the findings of this study [28].

Fracture gap after trauma was significant smaller in the non-operative group compared with the two operative groups. The size of displacement has always been important for the decision, whether patients should be operated or can be treated conservatively [3, 4]. Likewise, it is well accepted that residual steps are less tolerable than residual gaps [4, 25]. Already with a 5-mm gap and a 1-mm articular step [25], surgery should be considered.

From a prognostic point of view, fracture displacement of up to 5 mm could not be specified as minimally displaced. However, the aim of this study is to compare non-operative, ORIF and CRPIF treatment for acetabulum fractures. Several studies have shown, that highly displaced fractures (average initial displacement of 16 mm up to 30 mm) can be reduced adequately [23, 29, 31]. Therefore, a displacement of maximum 5 mm only represents minimally displaced fractures. This terminology has already been used in the literature [32].

It was shown that CRPIF can reduce blood loss and operative time significantly compared to ORIF. These findings are consistent with the previous literature [15, 16, 28]. Since short operative time and low blood loss are substantial, especially for geriatric patients with low cardiac reserve [33, 34], minimal invasive treatment options represent a conceivable alternative.

The length of hospital-stay and complication rates was low in patients treated non-operatively or by CRPIF. A long hospital stay is associated with prolonged bed rest resulting in cardiovascular, respiratory, musculoskeletal and psychological complications [6, 35].

Non-surgical complications occurred more likely with ORIF. The influence of the treatment method on the complication rate cannot be seen independent of hospital stay length, though. Performing a partial correlation to measure the impact of the different follow-up times, the complication rate after ORIF was no longer higher than after CRPIF and non-surgical treatment. But whether patients stayed in hospital due to their complications or complications occurred due to a prolonged hospitalisation cannot be told from the registry data available. Naturally, a more invasive surgery requires a longer monitoring of the patient.

There were no differences between open and percutaneous methods when looking at only surgical complications, however,—as this has been described for ORIF and CRPIF in the previous literature [28].

MRSA skin was captured as “non-surgical complication” since hospital-acquired MRSA colonialization occurs during hospital stay and may lead to serious MRSA infections especially in combination with surgical wounds [36].

There are several limitations concerning this study. First, registry studies do not allow for integration of variables not documented in the registry a priori, such as comorbidities, radiographic results of non-operative treatment at day of discharge, information about the weight-bearing area, indications for surgery and conservative treatment, postoperative management, weight-bearing and functional outcome. The range of age (60–100) is wide. Separating the patient population in 60–79-year-old and 80–100-year-old patients, the subgroups got too small for adequate statistical analysation (e.g., patients ≥ 80 years treated by CRPIF *n* = 19). Therefore, we can only make a limited guess and further research would be needed to specify the treatment methods in the context of geriatric age. We excluded fractures exhibiting a gap/step of more than 5 mm. Thus, our findings allow only conclusions for minimally displaced acetabulum fractures. Last, the follow-up time is limited to the hospital stay length and does not reflect late complications and the development of osteoarthritis. Differences may occur after discharge and, therefore, further studies comparing complications with long-term follow-up would be useful. It would be interesting to evaluate whether there is a difference in long-term complications depending on the residual displacement in different ages. Despite these limitations, this study presents data from a large multi-centric series on treatment of minimally displaced acetabulum fractures in the elderly.

Open reduction and internal fixation is the gold standards for young patients. In geriatric patients, the potential disadvantages of the higher invasiveness even of strictly intrapelvic or pararectus approaches must be considered.

Hence, conservative treatment has been described as a valid method in selected cases [5, 23, 37]. This study highlights a short hospital stay length and low rate of in hospital complications for non-operative treatment.

Percutaneous operative techniques, may pose a valuable alternative in geriatric patients that provides both: primary stability and minor surgical trauma. In this cohort of minimally displaced acetabulum fractures, CRPIF did not come at the cost of inferior quality of reduction. However, these results may not be generalised as radiographic outcome is not necessarily linked to functional outcome in all cases, especially in elderly patients. Individual considerations would be necessary for every patient. Minimal invasive methods are challenging and surgeons have to consider their own abilities [38]. Yet, imaging techniques are improving and knowledge is increasing, which will facilitate the application of these techniques in the future [30].

## Conclusion

Both operative treatments improve fracture displacement and joint congruency in elderly patients with minimally displaced acetabulum fractures. Compared to ORIF, CRPIF achieves similar quality of reduction but is associated with fewer complications, smaller intraoperative blood loss, shorter operative time and shorter length of hospital stay.
